# Embracing the informative missingness and silent gene in analyzing biologically diverse samples

**DOI:** 10.1038/s41598-024-78076-0

**Published:** 2024-11-16

**Authors:** Dongping Du, Saurabh Bhardwaj, Yingzhou Lu, Yizhi Wang, Sarah J. Parker, Zhen Zhang, Jennifer E. Van Eyk, Guoqiang Yu, Robert Clarke, David M. Herrington, Yue Wang

**Affiliations:** 1 Department of Electrical & Computer Engineering, Virginia Polytechnic Institute and State University, Arlington, VA 22203 USA; 2 Department of Electrical and Instrumentation Engineering, Thapar Institute of Engineering and Technology, Patiala, 147004 Punjab India; 3 Advanced Clinical Biosystems Research Institute, Cedars Sinai Medical Center, Los Angeles, CA 90048 USA; 4 Department of Pathology, Johns Hopkins University, Baltimore, MD 21231 USA; 5 Department of Automation, Tsinghua University, Beijing, 100084 P. R. China; 6 The Hormel Institute, University of Minnesota, Austin, MN 55912 USA; 7 Department of Internal Medicine, Wake Forest University, Winston-Salem, NC 27157 USA; 8 Dept. of Electrical and Computer Engineering, Virginia Polytechnic Institute and State University, 900 N. Glebe Road, Arlington, VA 22203 USA

**Keywords:** Computational biology and bioinformatics, Systems biology, Biomarkers

## Abstract

Bioinformatics software tools are essential to identify informative molecular features that define different phenotypic sample groups. Among the most fundamental and interrelated tasks are missing value imputation, signature gene detection, and differential pattern visualization. However, many commonly used analytics tools can be problematic when handling biologically diverse samples if either informative missingness possess high missing rates with mixed missing mechanisms, or multiple sample groups are compared and visualized in parallel. We developed the ABDS tool suite specifically for analyzing biologically diverse samples. Collectively, a mechanism-integrated group-wise pre-imputation scheme is proposed to retain informative missingness associated with signature genes, a cosine-based one-sample test is extended to detect group-silenced signature genes, and a unified heatmap is designed to display multiple sample groups. We describe the methodological principles and demonstrate the effectiveness of three analytics tools under targeted scenarios, supported by comparative evaluations and biomedical showcases. As an open-source R package, ABDS tool suite complements rather than replaces existing tools and will allow biologists to more accurately detect interpretable molecular signals among phenotypically diverse sample groups.

**Supplementary Information:**

The online version contains supplementary material available at 10.1038/s41598-024-78076-0.

## Introduction

High-throughput molecular expression profiling technologies provide the ability to comparatively study many genes or proteins expressed in biologically diverse samples (samples belonging to different phenotypic groups)^1^. An important but underappreciated issue in proteomics or gene expression analysis is how best to impute informative missingness that is often associated with signature genes with uneven missing rates in different groups and mixed missing mechanisms^2^. Among many data-driven imputation methods, the categorical information associated with informative missingness is often ignored^3^. Another essential and challenging task is to identify high quality signature genes that uniquely characterize the group of interest against the rest. Ideally, a signature gene among molecularly distinct groups would be either uniquely expressed or silent in the group of interest but in no others^4^. However, test statistics used by most existing methods do not satisfy exactly this signature definition and are theoretically prone to detecting imprecise signatures^5^. Furthermore, while a typical heatmap design is visually effective, the common reference origin for expression measurements is altered by the classical standardization, with zero-expression replaced by floating negative values for different genes. As a result, the color coding does not correctly reflect the relative quality among signature genes.

**Fig. 1:**
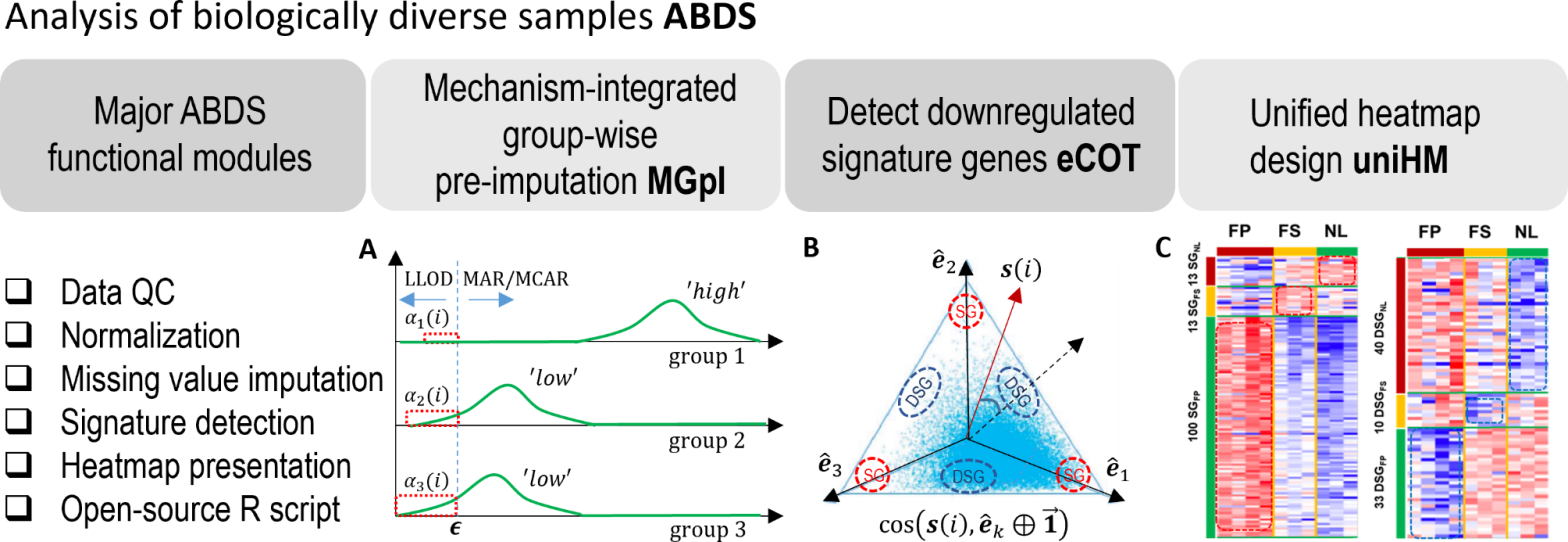
Overview of the modular ABDS tool suite with three analytics tools: MGpI, eCOT, and uniHM. (A) Illustrative intensity distributions of non-missing values over three groups, where signature gene expressions are high in group 1 (missingness dominated by MAR/MCAR) and low in groups 2 and 3 (missingness dominated by LLOD/MNAR). (B) Illustrative scatter simplex showing the referenced distributions of SGs and DSGs. (C) uniHM of the SGs and DSGs detected by COT/eCOT in real proteomics data.

Here we present ABDS tool suite assembled specifically for analyzing biologically diverse samples. Open-source R package includes three fundamental and interrelated analytic tools, namely, mechanism-integrated group-wise pre-imputation (MGpI), extended cosine-based one-sample test (eCOT)^5^, and unified heatmap design (uniHM). Collectively, we propose a hybrid imputation strategy to impute informative missingness associated with signature genes (SG), a cosine-score test to detect downregulated signature genes (DSG), and a unified heatmap design to comparably display multiple differential groups (Fig. 1). We demonstrate the effectiveness and utility of ABDS tools using both realistic simulations and real biomedical case studies, showing improved performance as compared with peer methods. The ABDS tool suite will allow biologists to more accurately detect true molecular signals from biologically diverse samples.

## Results

We evaluated the performance of MGpI and eCOT in comparison with representative or standard peer methods^5,6^. The evaluation does not include uniHM because it is a subjective visualization tool. We then conducted case studies to demonstrate the utility of these tools in biomedical applications. We used two quantitative measures to evaluate imputation accuracy, namely Root Mean Square Error (RMSE) and Normalized Root Mean Square Error (NRMSE). Specifically, RMSE and NRMSE are given by^7,8^

$$\:\text{R}\text{M}\text{S}\text{E}=\:\sqrt{\frac{\sum\:_{{\Omega\:}}{({\widehat{X}}_{{\Omega\:}}-{X}_{{\Omega\:}})}^{2}}{\left|{\Omega\:}\right|}},\:\text{N}\text{R}\text{M}\text{S}\text{E}=\:\sqrt{\frac{\sum\:_{{\Omega\:}}{({\widehat{X}}_{{\Omega\:}}-{X}_{{\Omega\:}})}^{2}}{\left|{\Omega\:}\right|{\sigma\:}_{{X}_{{\Omega\:}}}^{2}}},$$

respectively, where $\:{\Omega\:}$ is the index set of missing values in complete data matrix $\:X$, $\:\left|{\Omega\:}\right|$ is the total number of missing values, $\:\widehat{X}$ is the imputed complete data matrix, and $\:{\sigma\:}_{{X}_{{\Omega\:}}}^{2}$ is the variance of missing values. We used both partial Receiver Operating Characteristic (pROC) curve and the area under pROC (pAUC) to assess the accuracy of detecting silent signature genes.

### Experimental design and protocol

For real omics data, there is no method that can truly assess the accuracy of various imputation methods, because missing values will never be known and masked values cannot serve as the ground-truth missing values for unbiased evaluation^6,9^. While there are multiple causes for missing values in omics data, three typical missing mechanisms are widely acknowledged. For example, low abundant proteins or transcripts are easily missed because their concentration is below the lower limit of detection (LLOD); and poorly ionizing peptides may also cause proteins to be missing not at random (MNAR)^10^. However, missingness may also extend to mid- and even high-range intensities^11^, statistically categorized into missing at random (MAR)^12^. More precisely, MAR is missing conditionally at random and is associated with observed data distribution or underlying parametric covariates. While MAR allows prediction of the missing values based on observed data, unfortunately, the MAR and MNAR conditions cannot be distinguished based on the observed data because by definition missing values are unknown^9,13^. More importantly, missing values in reality can originate from a mix of both known and unknown missing mechanisms^12,14^.

To demonstrate the efficacy of MGpI, we evaluated and compared the accuracy of imputations by MGpI and seven peer methods on ground truth embedded realistic simulation data generated from two real omics data sets (LAD45 proteomics data^10^and Single-cell RNA Seq data^15^). To ensure a good balance between data quality and sample size, we used two guiding criteria for the sample selection: (i) relatively-balanced sample sizes across multiple groups, and (ii) sufficient data quality. We excluded data with non-informative missingness or high sample heterogeneity. For proteomics data, the realistic simulations involve 4 groups (normal – NL, fatty streaks – FS, fibrous plaques – FP, complex lesion – FC; pathologically-scored), 713 features with good quality, and 292 samples (imbalanced, 143 NL, 79 FS, 56 FP, 13 FC). Because human artery tissues are highly heterogeneous and the cross-group sample sizes are highly imbalanced, sample clustering was performed to remove those samples where their data deviated from the group-center with a higher than the acceptable threshold and select a subset of representative samples with more balanced group sizes (10 NL, 20 FS, 30 FP, 10 FC). There are 120 SGs (30 SGs per group) with cosine values of 0.7 ~ 0.95 ^5^. The ground-truth missing values were introduced and assigned by assigning some of the observed values with NA. Theoretically, any gene may contain some missing values. The introduced missing values are expectedly dominated by random missing mechanism in the group where SGs are highly expressed and dominated by LLOD in the groups where SGs are lowly expressed. Overall missing rates are 40 ~ 60% with MAR missing proportions of 30 ~ 50%.

For single-cell RNA Seq data, the realistic simulations involve five groups, 2,221 features, and 4,117 cells (imbalanced, cardiac muscle cell 133, endocardial cell 165, endothelial cell 1177, fibroblast 2119, leukocyte 523). Since the non-informative missingness rates are high and the cross-group sample sizes are highly imbalanced, samples were first filtered based on the zero-value ratio (< 400 out of 2,221 per sample), removing samples where non-informative missingness rates are higher than the acceptable threshold. We then used sample clustering to select a subset of representative samples with higher quality and cross-group balance (cardiac muscle cell 50, endocardial cell 20, endothelial cell 30, fibroblast 600, leukocyte 100). SGs are selected by COT (30 SGs per cell type). Masked missing values are introduced to non-zero values only with overall missing rate of 40%, 50% or 60%, of which 30%, 40% or 50% are MAR and remaining due to LLOD. Evaluation of imputation accuracy is measure over masked missing values only.

**Fig. 2:**
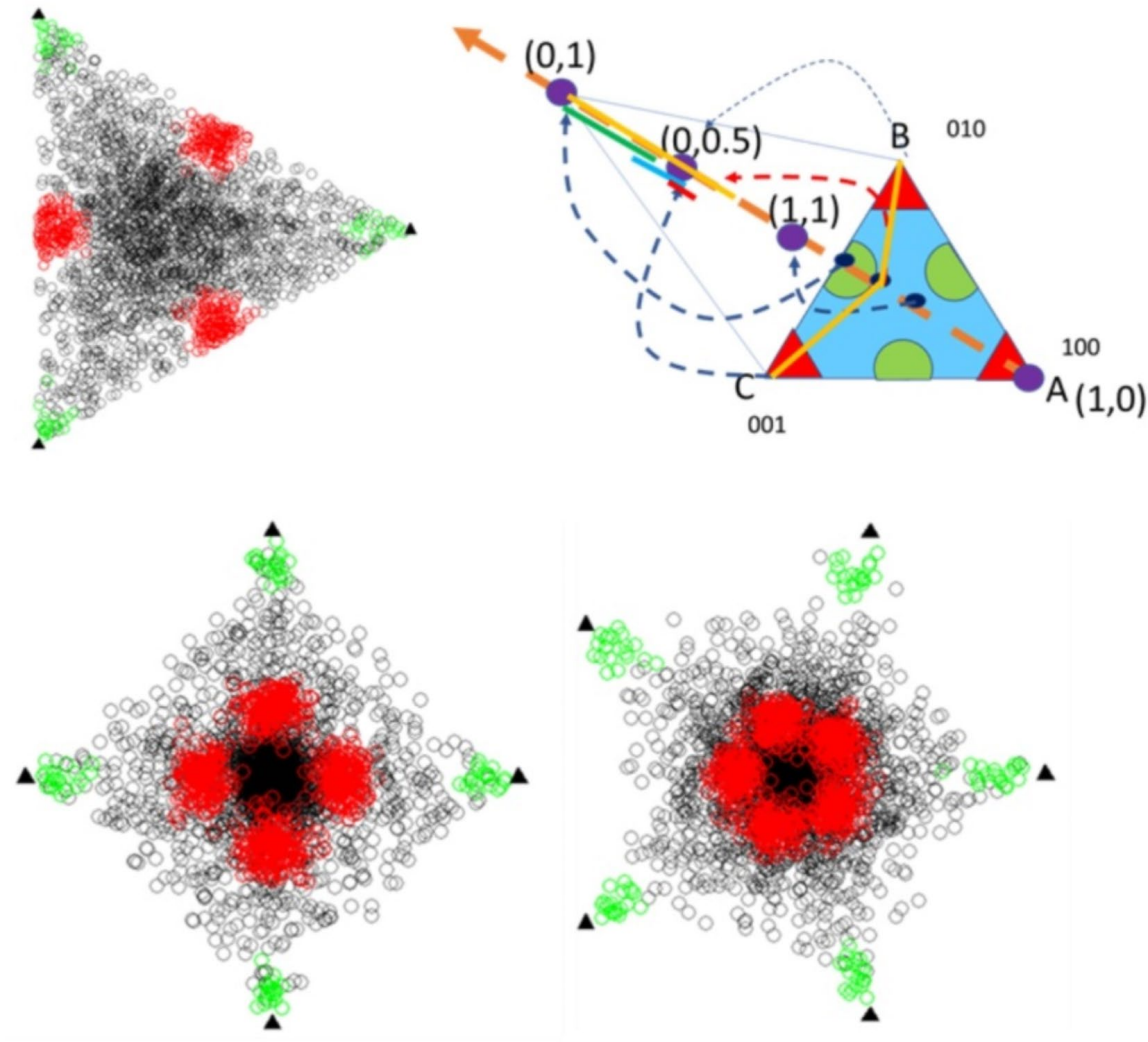
Scatter simplex of simulation data for assessing the performance of eCOT (including feature movement associated with the group merging BC versus A – multiphase airflow dynamics or fluid diffusion flow in OVR test), where red circles represent DSGs.

For assessing the accuracy of eCOT, the simulation data were generated according to the following design settings (see the scatter simplex illustrated in Fig. 2): *K* = 3 ~ 5 groups are considered, feature distribution under the null hypothesis (non-DSGs) follows the mixture of a symmetric Dirichlet distribution (1,200 features, black dot, $\:\alpha\:$=1), a Dirichlet distribution (1,200 features, black dot $\:\alpha\:$=4), and a truncated/non-negative zero-mean Gaussian distribution centered at simplex vertices (20 features/SGs per group, green dot); feature distribution under the alternative hypothesis (DSGs) follows a truncated/non-negative zero-mean Gaussian distribution centered at the centers of simplex facets (50 DSGs per group, red dot). Note that eCOT detects DSGs in *K*-dimensional space while OVR-FC/t-test works in one-dimensional scalar space after merging the rest into a single group (Fig. 2 illustrates the feature movement associated with the group merging – multiphase airflow dynamics or fluid diffusion flow).

### Comparative evaluation of MGpI on realistic simulation data

We evaluated the accuracy of MGpI in comparison with seven representative peer methods on ground truth embedded realistic simulation data generated from two benchmark omics data sets (LAD45 proteomics data^10^and Single-cell RNA Seq data^15^). Our experiments emphasized SGs because these genes typically exhibit high and uneven missing rates or mechanisms across different groups. The introduced missing values are dominated by random missing mechanism in the group where SGs are highly expressed and dominated by LLOD in the groups where SGs are lowly expressed (Fig. 1A).

We imputed missing values based on equation #1. We used both the root mean squared error (RMSE) and normalized RMSE (NRMSE) between the imputed value $\:\stackrel{\sim}{\varvec{x}}\left({i}_{\text{SG}}\right)$ and the ground truth $\:\varvec{x}\left({i}_{\text{SG}}\right)$to assess imputation accuracy^6^. The experimental results show that MGpI consistently outperforms all seven peer methods with lower RMSE and NRMSE on both general features and SGs in these experiments (Tables 1 and 2, **Tables S1-S2**). It can be seen that the relative performance of various imputation methods varies between proteomics and single-cell RNA Seq data. This should be expected because different data types have different yet complex missingness patterns. It should be noted that the ability to simulate the missing values mechanisms (MNAR, MAR) depends on the efficacy of the tools applied. While it may be informative to compare the impact of the imputation versus non-imputation on some subsequent data analysis, we have opted to focus on assessing direct imputation accuracy using realistic simulations with available ground truth, because the evaluation using subsequent analysis would be indirect and task-dependent.

**Table 1:**
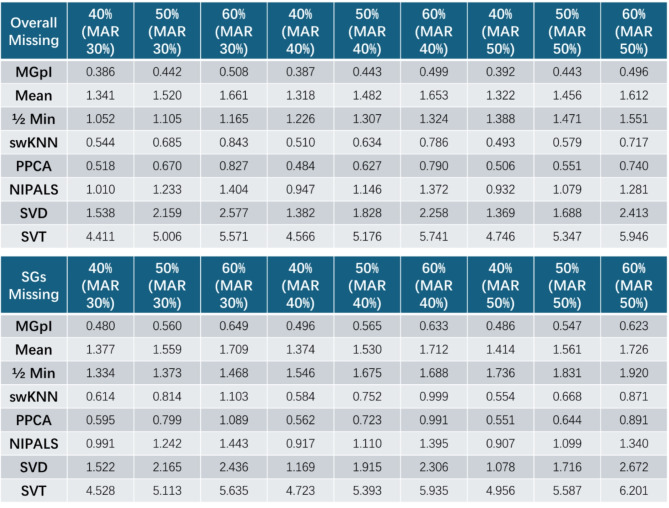
Imputation accuracy achieved by MGpI compared with seven peer methods on realistic simulation data (LAD45 proteomics data) embedded with ground truth and measured by RMSE (overall and SGfocused).

**Table 2:**
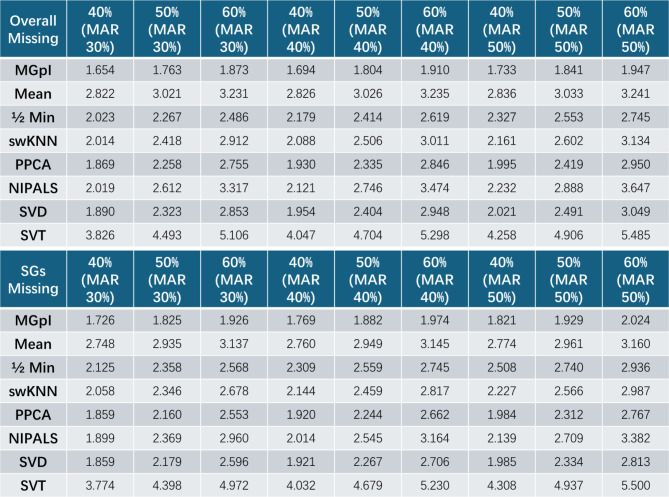
Imputation accuracy achieved by MGpI compared with seven peer methods on realistic simulation data (single-cell RNA Seq data of heart tissue) embedded with ground truth and measured by RMSE (overall and SG-focused).

### Comparative evaluation of eCOT-DSG on simulation data

**Fig. 3:**
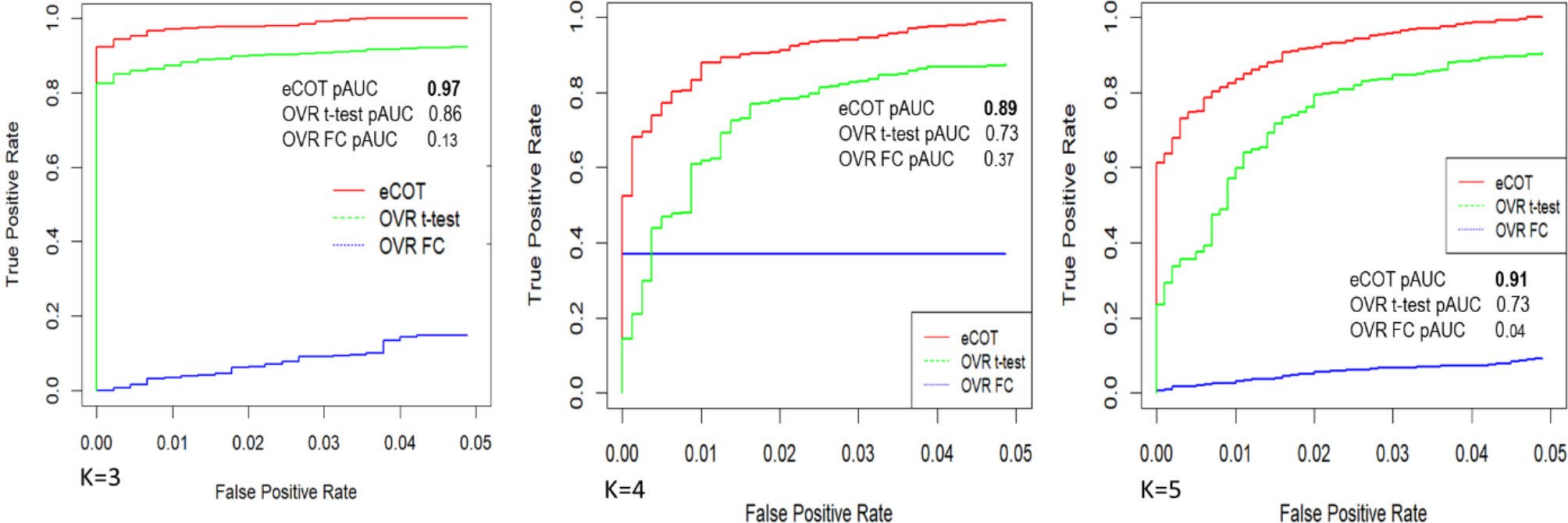
Detection accuracy of DSGs achieved by eCOT compared with benchmark OVR t-test and OVR-FC test on simulation data embedded with ground truth, measured by pROC curves and pAUC values at false positive rate of 0.05.

We evaluated the accuracy of eCOT-DSG in comparison with two most relevant and suitable benchmark methods, namely One Versus Rest t-test (OVR t-test) and One Versus Rest Fold Change (OVR-FC), on ground truth embedded simulation data^5^. In our previous report on the COT framework for detecting SGs, we have compared the performance of COT-SG with additional methods such as ANOVA and Limma/EdgeR. Here we opted not to include ANOVA and Limma/EdgeR in the comparison because they are not designed for detecting DSGs. Simulation data include general genes generated from a mixture of two Dirichlet distributions, and realistic SGs and DSGs (Fig. 1B, Supplementary Information). We used both partial Receiver Operating Characteristic (pROC) curve and the area under pROC (pAUC) to assess DSG detection accuracy. The experimental results show that eCOT consistently outperforms the two benchmark methods with higher pAUC and almost perfect power at standard false positive rate cutoff for *K* = 3, 4, 5 (Fig. 3).

The null distribution plays a crucial role in large-scale multiple testing when false positives are of great concern. However, because the number of pure group samples is often very small and non-DSG patterns are often highly complex and intrinsically data-dependent, classical schemes to estimate the null distribution in a two/multiple-sample test setting is impractical^16^or even inappropriate^17^. A reasonable assumption is that the observed data can show the null distribution when a significant majority of features are associated with the null hypothesis^17^.

### Interpretable biomedical case study

**Fig. 4:**
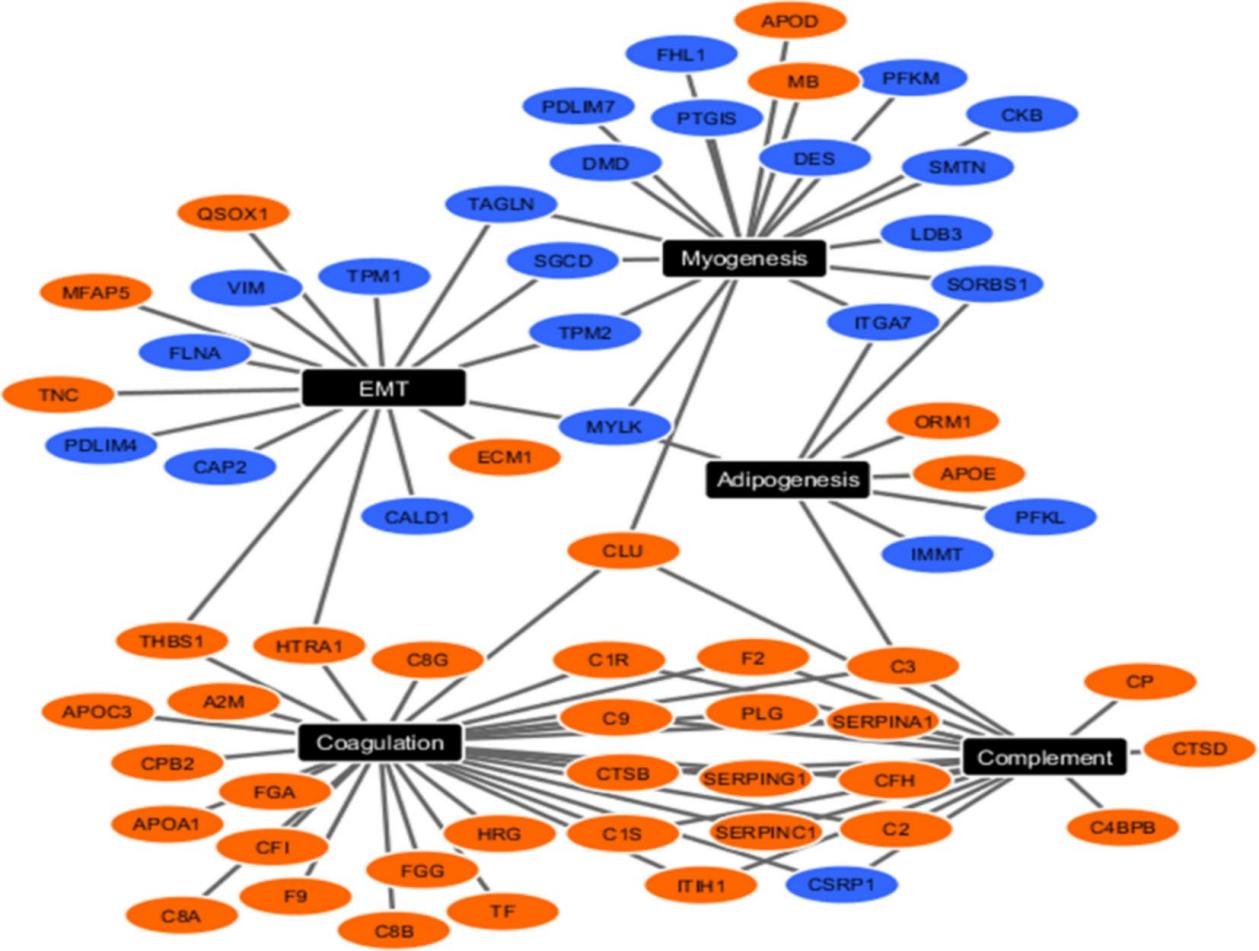
Upregulated (orange nodes) and downregulated (blue nodes) SGs/DSGs detected by COT/eCOT in FP group clustered into the top 5 functional pathways from the MSigDB component of Enrichr pathway analysis software.

We applied eCOT to a proteomics dataset acquired from human artery samples (targeted pure specimens) highly-enriched by the tissue types associated with atherosclerosis in the tissue-based validation phase^10,18^. Samples were divided into three phenotypically ‘pure’ groups based on the severity of atherosclerosis pathogenesis (100% FP, *n* = 4; 100% FS, *n* = 3; 100% NL, *n*= 3)^18^. We surveyed all cosine scores of group-averaged super-samples and reported top SGs and DSGs (Fig. 1C, **Table S3-S4**). Functional pathway analysis of tissue type-specific SGs and DSGs produced results consistent with known pathogenesis in atherogenesis. Network analysis of the top enriched functional pathways associated with FP showed that SGs were enriched for complement and coagulation functions, whereas DSGs were enriched for myogenesis and EMT (Fig. 4, **Figure S2**). Together, this pattern is consistent with the increased inflammation and decreased smooth muscle cell contractile phenotype composition seen in atherosclerotic lesions. Since IL2-related DSGs were enriched in the NL, this finding could reflect that lower IL2 signaling is protective against atherosclerotic plaque development^18–21^. While equal numbers of SG and DSGs were confidently identified for the FP group, there was lower SG quality (e.g., lower cosine score) and fewer SG numbers identified for the FS and NL groups, and in contrast DSGs for these groups were strong. Pathway analysis for the FS and NL groups indicated marker genes associated with mTORC1 signaling and reactive oxygen species (ROS) pathway enriched among FS signature proteins and myogenesis, EMT, hypoxia and IL2/STAT5 signaling were enriched among NL signature proteins (**Fig. S2**). Both mTORC1 and ROShave previously been linked to atherogenesis^22,23^. Interestingly, IL2 in blood vessels is produced, at least in part, by resident T cells with IL2 receptors located on the smooth muscle cells^24^and IL2 signaling has been linked to atherogenesis^25^. Since IL2-related proteins were enriched among the DSG in the NL group, this finding could reflect that lower IL2 signaling is protective against atherosclerotic plaque development. This hypothesis and others generated from the analysis of SG and DSG warrants further testing in future studies.

We also checked the suitability of sample sizes in relation to power expectation based on simplified and practical power analysis guidelines^26,27^. For the power calculation, we considered the simplest case of comparing cosine scores of super-sample profiles across the three discrete pathologic groups using the ANOVA framework^26^. With the one-sample test in eCOT using 3 ~ 4 ‘pure’ samples per group, assuming a Cohen’s effect-size of 2 and a 0.05 significance level, we estimated a power of 80% (Table 4 in^26^). A simpler formula also estimated 4 ‘pure’ samples per group in our study (*K*= 3, Table 5 in^26^).

### Visualizing expression patterns of SGs/DSGs by uniHM

We used the newly designed heatmap to display the differential expression patterns of the DSGs reported in Sect. 3.3 (Fig. 1C), in comparison with the classically designed heatmap (**Fig. ****S1**). Using this newly implemented heatmap function, DSGs are arranged based on their sample-averaged cosine scores with respect to hypothesis-enumerated references. The new heatmap visually reflects the idealness of DSGs where the common origin remains the same across all genes and the contrast is consistent with the corresponding cosine scoring.

### Additional biomedical case study using eCOT-DSG

We applied eCOT to our Edinburgh breast cancer gene expression data from mostly estrogen receptor-positive tumors acquired prior to standard endocrine therapy. Samples were divided into four roughly equal-sized phenotypic groups based on the follow-up sample-wise recurrence times. We again surveyed and reported all SGs/DSGs (**Figs. S3-S4**, **Tables S5-S6**). Signaling activated downstream of EGFR family members is a central feature of breast cancer. HER2/ERBB2 is the most widely studied, where protein overexpression or gene amplification defines one of the three primary breast cancer groups and targeting the HER2 protein and/or blocking its kinase function greatly improves overall survival is now standard of care for patients with HER2 + breast tumors. When applied to transcriptome data from breast cancer patients, the eCOT identified EGFR/ERBB2 and multiple EGFR-related downstream targets as enriched in estrogen-receptor positive (ER+) breast cancers likely to recur late (≥ 5 years after initial diagnosis). Most of these tumors were treated with the antiestrogen Tamoxifen and many patients would experience an overall survival benefit from Tamoxifen. However, consistent with the eCOT prediction, higher expression of EGFR (ERBB) or HER2 (ERBB2) would be expected to reduce Tamoxifen responsiveness and increase the likelihood of a subsequent recurrence. Unfortunately, the unknown effect-size does not support a formal power analysis.

## Discussion

ABDS suite presents three novel data analytics tools for analyzing biologically diverse samples across multiple groups. These tools are specifically designed to complement existing methods for imputing mechanism-mixed informative missingness, detecting downregulated signature genes, and visualizing complex differential expression patterns. Specifically, MGpI enables recruiting critical SGs that are often prematurely eliminated due to high overall missing rates. Moreover, the detected DSGs will allow researchers to study silenced pathways, assisting potentially more comprehensive characterization of disease progression. For readers interested in the relevant mathematical formulation and algorithmic workflow, we highly recommend the original reports^4–6^. While the focused applications here involve gene or protein expression data acquired from bulk or sorted-cell samples, the ABDS tools are principally generalizable to other molecular omics measurements with further developments.

We emphasize that the ABDS suite is intended to complement rather than replace existing tools. For example, MGpI imputes potentially informative missingness associated with signature genes that may be eliminated prematurely due to relatively high overall missing rates. The key difference between MGpI and existing methods is that MGpI performs group-wise imputation by considering both MNAR and MAR/MCAR mechanisms within each group, thus serving as a pre-imputation step. We note that MGpI may lose some power due to smaller within-group sample size. Hence, we recommend that users may apply global methods to refine missing value imputation using all samples after MGpI^3,6^. We also advise users to apply a classical heatmap design for visualizing differential expression patterns.

Based on the SGs detected in our atherosclerosis case study, preliminary results have identified several highly promising regulatory molecules including transcription factors (TFs) (e.g., SOX9, SPI1, TCF4, PGR, FOXO4) and non-coding RNAs (e.g., linc1503, miRNA let-7e-5p) as strongly linked to the expression of proteomic SGs indicating initiation or progression of disease. Manipulation of these molecules could be exploited for early therapeutic intervention. Our preliminary data also highlight specific SG-enriched cell-types (adventitial nerve cells, pericytes) and suppression of several canonical markers of the vascular smooth muscle cell (VSMC) contractile phenotype (e.g., CNN1, SMTN^28^). These data are consistent with mounting evidence of adventitial neuro-immune interactions contributing to the pathogenesis of atherosclerosis^29^. These interactions may be uniquely responsible for expression of potentially pathogenic proteomic SGs in specific cell types^20,30^ and could reflect opportunities for the development of cell-type specific interventions.

## Method

### Mechanism-integrated group-wise pre-imputation

Signature genes play important roles in studying and characterizing biologically diverse samples^18^. Missing values associated with these genes are expected to have a group-specific mix of different missing mechanisms and cross-group uneven missing rates. Thus, using an overall missing rate for data quality control would be problematic and could adversely affect subsequent analyses. For example, the current practice in analyzing omics data containing missing values is to eliminate genes with overall missing rates higher than a threshold. This would not be ideally applicable to biologically diverse samples, e.g. belonging to multiple yet different groups. A common solution for missingness is to impute the missing values based on assumed missing mechanisms. However, this approach can introduce a profound change in the distribution of protein-level intensities because most methods are only designed for a single missing mechanism^2^. These changes can have unpredictable effects on downstream differential analyses. For example, MNAR in the group(s) dominated by LLOD is often imputed in the same way as in the groups dominated by MAR mechanisms^6^, ignoring the categorical information about biologically diverse samples.

We propose a mechanism-integrated group-wise pre-imputation (MGpI) strategy that explicitly considers mixed missing mechanisms varying across different phenotypic groups, where we assume that the molecular expression data are approximately and normally distributed. First, with an initial data normalization based on a subset of genes with no missingness, a common overall minimum value ε associated with LLOD is determined from all observed values of the full data matrix in log-space. Second, for each gene *i* and for each group *k*, group-specific mean value $\:{\stackrel{-}{x}}_{k}\left(i\right)$ and standard deviation $\:{\sigma\:}_{k}\left(i\right)$ are calculated. Note that none of missing values (NA) is involved in the estimation of these model parameters. Third, within each group *k*, a missing value is imputed by

1 $$\:\begin{array}{c}{\stackrel{\sim}{x}}_{k}\left(i\right)={\alpha\:}_{k}\left(i\right)\epsilon/2+\left[{1-\alpha\:}_{k}\left(i\right)\right]{\stackrel{-}{x}}_{k}\left(i\right)\end{array}$$

where $\:{\alpha\:}_{k}\left(i\right)$ is the probability of LLOD missing mechanism (the area under the green curve outlined by the red-dash block in Fig. 1A) and is estimated by ε and the approximated normal distribution specified by $\:{\stackrel{-}{x}}_{k}\left(i\right)$ and $\:{\sigma\:}_{k}\left(i\right)$. MGpI scheme integrates two popular and simple imputation methods^2,6^, i.e., weighted ‘overall min/2’ for imputing LLOD/MNAR missingness and ‘group-specific mean’ for imputing MAR/MCAR missingness. Specifically, for each group *k*, we plug-in the overall minimum observed value to the estimated normal distribution to determine the probability $\:{\alpha\:}_{k}\left(i\right)$ of LLOD/MNAR (under green curve within red block), then assign $\:\left[{1-\alpha\:}_{k}\left(i\right)\right]$ to be the probability of MCAR/MAR.

### Seven most-relevant peer methods for missing value imputation

- **min/2**(half minimum): Taking MNAR as the missing mechanism (e.g. LLOD), for each protein the missing values are estimated as half the minimum value of the observed intensities in that protein across all samples^9,10^.
- **Mean**: For MAR/MCAR as the missing values mechanism, for each protein we replaced the missing values with the mean value of the observed intensities in that protein across all samples^9,10^.
- **swKNN**(sample-wise k-nearest neighbors): Taking MAR as the missing values mechanism, we leveraged local similarity among samples for each protein, replacing the missing values with the weighted average of observed intensities in that protein proportional to the proximities of k-nearest neighboring samples^9^.
- **PPCA**(probabilistic PCA): For MCAR/MAR as the missing values mechanism, a low-rank probabilistic PCA matrix factorization was estimated by the expectation maximization (EM) algorithm and then used to impute missing values^31^.
- **NIPALS**(non-linear estimation by iterative partial least squares): Taking MCAR/MAR as the missing values mechanism, a low-rank missing-data-tolerant PCA matrix factorization was estimated by iterative regression and then used to impute missing values^32,33^.
- **SVD**(SVDImpute): For MCAR/MAR as then missing values mechanism, a low-rank SVD matrix factorization was estimated by the EM algorithm and used to impute missing values^32,34^.
- **SVT**(singular value thresholding): Where we assumed MCAR/MAR to be the missing values mechanism, a low-rank SVT matrix factorization was estimated by iteratively solving a nuclear norm minimization problem and then used to impute missing values^35^.

### Extended cosine-based one-sample test on downregulated signature genes

An important but frequently underappreciated issue is how best to define and detect a cell or tissue marker among many groups. Here we extended cosine-based one-sample test (COT), a SG detection method that we previously developed^5^. For readers interested in the mathematical formulation, algorithmic workflow, and comparative evaluations of the COT approach for detecting SGs, we highly recommend the original reports^5,16^.

In addition to signature genes^4,5^, a molecularly distinct group may also be characterized by features that are uniquely silent in the group of interest but in no others (Fig. 1B), i.e. the aforementioned DSGs. Mathematically, a DSG of group *k* is defined,

2 $$\:\begin{array}{c}\left\{\begin{array}{c}{x}_{k}\left({i}_{\text{DSG,}\text{k}}\right)\approx\:0,\\\:{x}_{l\ne\:k}\left({i}_{\text{DSG,}\text{k}}\right)\gg\:0,\end{array}\right.\end{array}$$

where $\:{x}_{k}\left({i}_{\text{DSG,}\text{k}}\right)$ and $\:{x}_{j\ne\:k}\left({i}_{\text{DSG,}\text{k}}\right)$ are the average expressions of DSG $\:{i}_{\text{DSG,}\text{k}}$ in groups *k* and $\:j$, respectively. However, test statistics used by most existing methods do not satisfy exactly this DSG definition and are theoretically prone to detecting imprecise DSGs^5^. The most frequently used methods rely on an ANOVA model that adopts the null hypothesis that samples in all groups are drawn from the same population and is originally designed to detect differentially-expressed genes across any of the groups. Another popular method is the One-Versus-Rest Fold Change or t-test (OVR-FC/t-test/Limma/EdgeR) that is based on the ratio of the averaged expression in a particular group to the averaged expression in all other groups^36,37^. However, a gene with a low average expression value in the rest is not necessarily expressed at a low level in every group in the rest.

According to (2), the cross-group expression pattern of an ideal DSG can be represented concisely by the vector $\:{\widehat{\varvec{e}}}_{k}\oplus\:\overrightarrow{1}$ (one-zero degenerate $\:\overrightarrow{1}$), where $\:{\widehat{\varvec{e}}}_{k}$ are the Cartesian unit vectors, $\:\overrightarrow{1}$ is the all-1s vector, and $\:\oplus\:$ is the exclusive disjunction XOR operation on the Cartesian unit vectors $\:{\widehat{\varvec{e}}}_{k}$, readily serving as a reference for a one-sample test. Conceptually, the null hypothesis for non-DSG, and the alternative hypothesis for DSG, can be described as

3 $$\:\begin{array}{c}\begin{array}{c}{H}_{\text{non-}\text{DSG}}^{\text{n}\text{u}\text{l}\text{l}}:\:\:\:\:\:\:\:\varvec{x}\left(i\right)\ne\:{\widehat{\varvec{e}}}_{k}\oplus\:\overrightarrow{1};\\\:{H}_{\text{DSG}}^{\text{alt}\text{ernative}}:\:\:\:\varvec{x}\left(i\right)={\widehat{\varvec{e}}}_{k}\oplus\:\overrightarrow{1};\end{array}\end{array}$$

where $\:\varvec{x}\left(i\right)=\left[{x}_{1}\left(i\right),\:{x}_{2}\left(i\right),\:\dots\:,\:{x}_{K}\left(i\right)\right]$ is the sample-averaged cross-group expression vector of gene *i*, and *K* is the number of groups. Fundamental to the success of eCOT is the magnitude-invariant test statistic $\:\text{cos}\left(\varvec{x}\left(i\right),\:{\widehat{\varvec{e}}}_{k}\oplus\:\overrightarrow{1}\right)$ that measures the match between the cross-group expression pattern $\:\varvec{x}\left(i\right)$ of gene *i* and the ideal DSG expression pattern of constituent groups in scatter space (Fig. 1B)

4 $$\:\begin{array}{c}{t}_{\text{eCOT}}\left({i}_{\text{DSG,}\text{k}}\right)=\text{cos}\left(\varvec{x}\left(i\right),\:{\widehat{\varvec{e}}}_{k}\oplus\:\overrightarrow{1}\right),\end{array}$$

where $\:1/\sqrt{K-1}<{t}_{\text{eCOT}}\left(i\right)<1$ (Supplement Information). Under the assumption that most genes are associated with the null hypothesis, eCOT approximates the null distribution with the empirical histogram of the test statistics estimated directly from the data.

### Unified heatmap design for comparative display

A popular heatmap design for displaying differentially expressed genes is to standardize each gene separately, that is, the expression levels of each gene across samples are first centered and then normalized by standard deviation. To address the aforementioned drawbacks, we now propose an alternative heatmap design that can display the differential patterns among multiple groups consistent with the quality of SGs/DSGs. Specifically, for each gene *i*, the sum of group-specific mean values is calculated and used to normalize the expression level $\:x\left(i\right)$ in individual samples in linear space

5 $$\:\begin{array}{c}\widehat{x}\left(i\right)=x\left(i\right)/{\sum\:}_{k=1}^{K}{x}_{k}\left(i\right),\end{array}$$

where $\:\widehat{x}\left(i\right)$ is the perspective projection of $\:x\left(i\right)$ onto a scatter simplex. The proximity of normalized cross-group expression vectors $\:\widehat{\varvec{x}}\left(i\right)$to the signature references reflects the quality of SGs/DSGs, measured by the corresponding cosine values^5^. The group-specific mean value $\:{\widehat{x}}_{k}\left(i\right)$ and standard deviation $\:{\widehat{\sigma\:}}_{k}\left(i\right)$ are then calculated in log-space and used to standardize the expression values of SGs/DSGs for display purpose. Furthermore, we can order each sample or gene based on their sample/gene-averaged cosine values with respect to SG/DSG references (Fig. 1C) (Supplementary Information).

We should clarify that in the uniHM design, between-sample normalization in linear-space remains a prerequisite for any downstream analysis, by creating a comparable gene-wise distribution across samples or groups. Standardization in log-space is for display purpose to ensure comparable contrast across genes. Importantly, our new design can visually rank the discriminatory genes in relation to a common color-coded origin across all genes.

### ABDS software package

The ABDS tool suite consists of three unique yet interrelated analytics tools (Fig. 1) implemented in R package. A user’s guide and a vignette are provided. The software packages are evaluated by community-trial software testing. The R package is open-source at GitHub, and is distributed under the MIT license. The ABDS software tools are easy to use and principally applicable to other omics data with further development. Group label on each sample is required. The output file contains the cosine scores for individual genes with respect to the ideal SG/DSG references.

## Electronic supplementary material

Below is the link to the electronic supplementary material.

Supplementary Material 1

## Data Availability

The R package of ABDS tool suite is freely available at https://github.com/niccolodpdu/ABDS. The package is developed based on R version 4.3.1 https://www.r-project.org/. The operation system can be any system supporting R language. Human artery proteomics dataset was obtained from publicly available datasets from previously published study available at https://pubs.acs.org/doi/10.1021/acs.jproteome.0c00118 .
