# Supplementary material for: Embracing the informative missingness and silent gene in analyzing biologically diverse samples
Source: Sci Rep. 2024 Nov 16;14:28265. doi: 10.1038/s41598-024-78076-0 (PMC11569126; doi:10.1038/s41598-024-78076-0)
Supplement: Supplementary file 1 — Supplementary Material 1 [file 41598_2024_78076_MOESM1_ESM.docx]

Supplementary Information

**ABDS: a bioinformatics tool suite for analyzing biologically diverse samples**

Dongping Du, Saurabh Bhardwaj, Yingzhou Lu, Yizhi Wang, Sarah J. Parker, Zhen Zhang, Jennifer E. Van Eyk, Guoqiang Yu, Robert Clarke, David M. Herrington, Yue Wang

**Contents**

[Methods 2](#_Toc160173006)

[Results (with supplementary figures and tables) 4](#_Toc160173007)

[Discussion 11](#_Toc160173008)

[R Scripts 12](#_Toc160173009)

[References 13](#_Toc160173010)

## Methods

**Mechanism-integrated group-wise pre-imputation (MGpI)**

In relation to the existing mean imputation strategy, the major issue in classical mean imputation is that global mean would bias groups differences toward the null, and this bias may be more pronounced if there is asymmetry between the number of samples in each group.  The same effect can occur if group differences vary by batch.  Consider a toy example: 50% missingness, observed mean values FP=4, FS=2, NL=2; the global mean is 8/3; then the imputed mean values FP=3.33, FS=2.33, NL=2.33. For another gene (more differential), the observed mean values FP=4, FS=1, NL=1; the global mean is 2; the imputed mean values FP=3, FS=2, NL=2. In contrast, instead of using global mean, SGpI uses group-specific mean (together with min/2) to impute group-specific missing values. In the toy example, group-specific means imputed by MGpI remain unchanged.

**Review on previous COT for detecting signature genes**

As aforementioned, eCOT is an extended version COT. Here we introduce and discuss the original concept of COT for detecting SGs. Mathematically, an ideal SG of group *k* is defined as a gene expressed only in group *k* but not in any other groups (Chikina, et al., 2015; Delaney, et al., 2019; Kuhn, et al., 2011; Lu, et al., 2022), approximately

$$\begin{aligned} \left\{ \begin{aligned} s_{k}\left( i_{\text{SG}\text{,}\text{k}} \right)\gg0, \\ s_{l\neq k}\left( i_{\text{SG}\text{,}\text{k}} \right)\approx0, \end{aligned} \right.\boldsymbol{\#}\left( 1 \right) \end{aligned}$$

where $s_{k}\left( i_{\text{SG}\text{,}\text{k}} \right)$ and $s_{l\neq k}\left( i_{\text{SG}\text{,}\text{k}} \right)$ are the average expressions of marker gene $i_{\text{SG}\text{,}\text{k}}$ in groups *k* and $l$, respectively. We acknowledge that there are alternative definitions but in the absence of a universally accepted standard in the field, our definition provides some unique advantages to guide our work. We emphasize that group-specific SG as defined here are enriched uniquely in a particular group, regardless of their expression level(s), and their identities can be readily used in facilitating deconvolution or classification (Wang, et al., 2016).

Accordingly, the cross-group expression pattern of an ideal SG can be represented concisely by the Cartesian unit vectors ${\hat{\boldsymbol{e}}}_{k}$, readily serving as a reference for a one-sample test. Conceptually, the null hypothesis for non-SG, and the alternative hypothesis for SG, can be described as

$$\begin{aligned} \begin{matrix} H_{\text{non-}\text{SG}}^{\mathrm{null}}: \boldsymbol{s}\left( i \right)\neq{\hat{\boldsymbol{e}}}_{k}; \\ H_{\text{SG}}^{\text{alternative}}: \boldsymbol{s}\left( i \right)={\hat{\boldsymbol{e}}}_{k}; \end{matrix}\boldsymbol{\#}\left( 2 \right) \end{aligned}$$

where $\boldsymbol{s}\left( i \right)=\left[ s_{1}\left( i \right), s_{2}\left( i \right), \ldots, s_{K}\left( i \right) \right]$ is the sample-averaged cross-group expression pattern of gene *i*. Fundamental to the success of COT is the newly-proposed test statistic $\cos\left( \boldsymbol{s}\left( i \right), {\hat{\boldsymbol{e}}}_{k} \right)$ that measures directly the similarity between the cross-group expression pattern $\boldsymbol{s}\left( i \right)$ of gene *i* and the ideal SG expression pattern of constituent groups in scatter space given by

$$\begin{aligned} t_{\text{COT}}(i_{\text{SG}})=\underset{1\leq k\leq K}{\mathrm{argmax}} \cos\left( \boldsymbol{s}\left( i \right), {\hat{\boldsymbol{e}}}_{k} \right)=\underset{1\leq k\leq K}{\mathrm{argmax}}\frac{s_{k}\left( i \right)}{\sqrt{\sum_{j=1}^{K} \left[ s_{j}(i) \right]^{2}}},\#\left( 3 \right) \end{aligned}$$

where *K* is the number of constituent groups. Sample normalization and batch effect adjustment are the required preprocessing steps prior to COT analysis; when applicable, the input of COT should be a sample-normalized and batch-adjusted data matrix.

We previously implemented the COT workflow in both Python and R, and used community-based trials to test the COT software. The Python package is open-source at GitHub, built using NumPy and Pandas, and is distributed under the MIT license. The COT software tool is easy to use and applicable to multi-omics data. The rows of input data matrix correspond to genes or other molecular features, and the columns correspond to samples. The group label on each sample is required by the COT test statistic. The output file stores the input genes and their cosine values in reference to the ideal SG of respective groups (Lu, et al., 2022).

## Results (with supplementary figures and tables)

**Table S1**. Imputation accuracy achieved by MGpI compared with seven peer methods on realistic simulation data (LAD45 proteomics) embedded with ground truth and measured by NRMSE: A. All features, B. SG-focused.


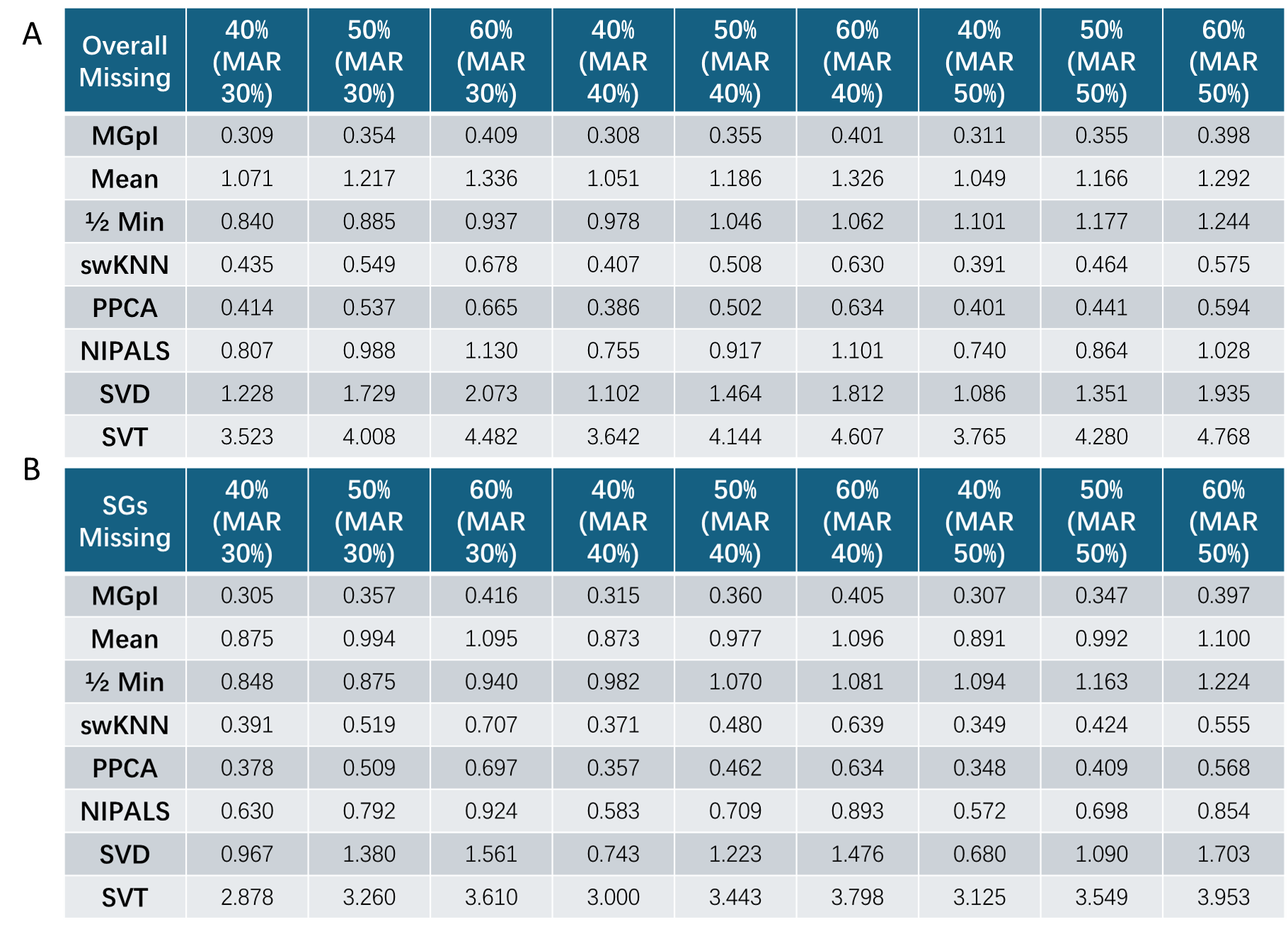


**Table S2**. Imputation accuracy achieved by MGpI compared with seven peer methods on realistic simulation data (single-cell RNA seq, heart tissue) embedded with ground truth: A. SG high expressed group measured by RMSE; B. SG-focused measured by NRMSE.


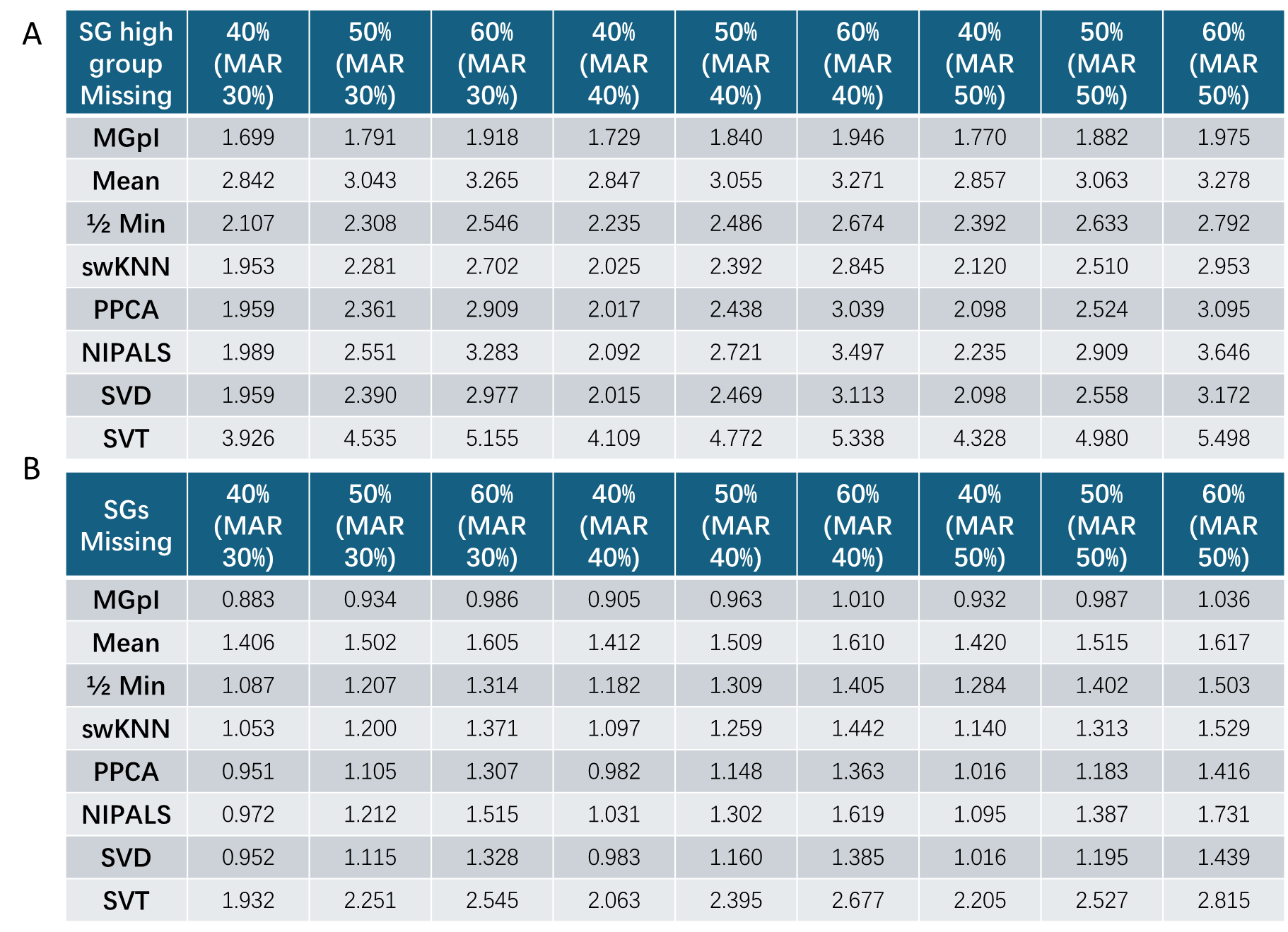


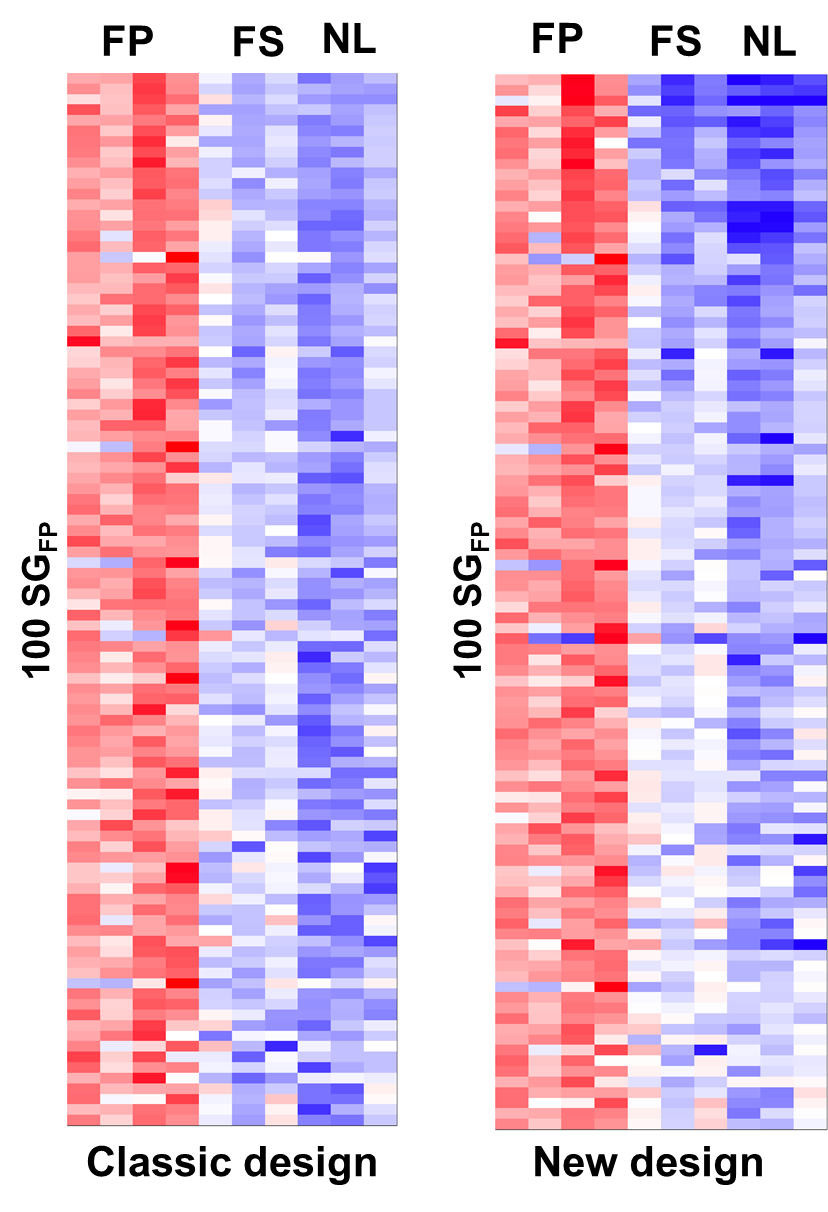


**Figure S1.** A side-by-side display of comparing the classic heatmap and the new heatmap uniHM, on the 100 protein SGs associated with FP, where the quality of SGs are ranked by the cosine values of their cross-group expressions in relation to the reference expression pattern (top-down).


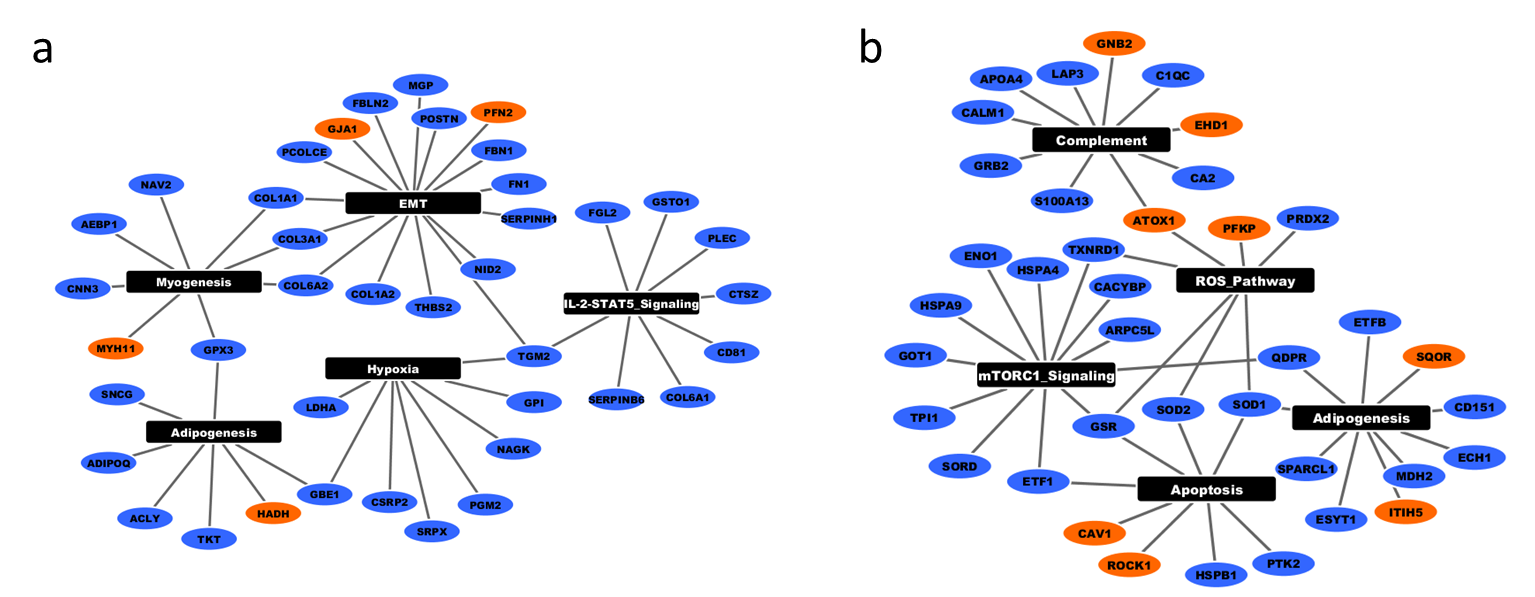


**Figure S2**. SGs (orange nodes) and DSGs (blue nodes) signature genes clustered into the top 5 functional pathways from the MSigDB component of Enrichr pathway analysis software (black nodes) are shown for the normal NL (A) and fatty streak FS (B) groups. Together, this pattern is consistent with the increased inflammation and decreased smooth muscle cell contractile phenotype composition seen in atherosclerotic lesions. Pathway analysis indicated mTORC1 signaling and reactive oxygen species pathway as enriched in FS and myogenesis, EMT, hypoxia and IL2/STAT5 signaling in NL, as previously been linked to atherogenesis.

**
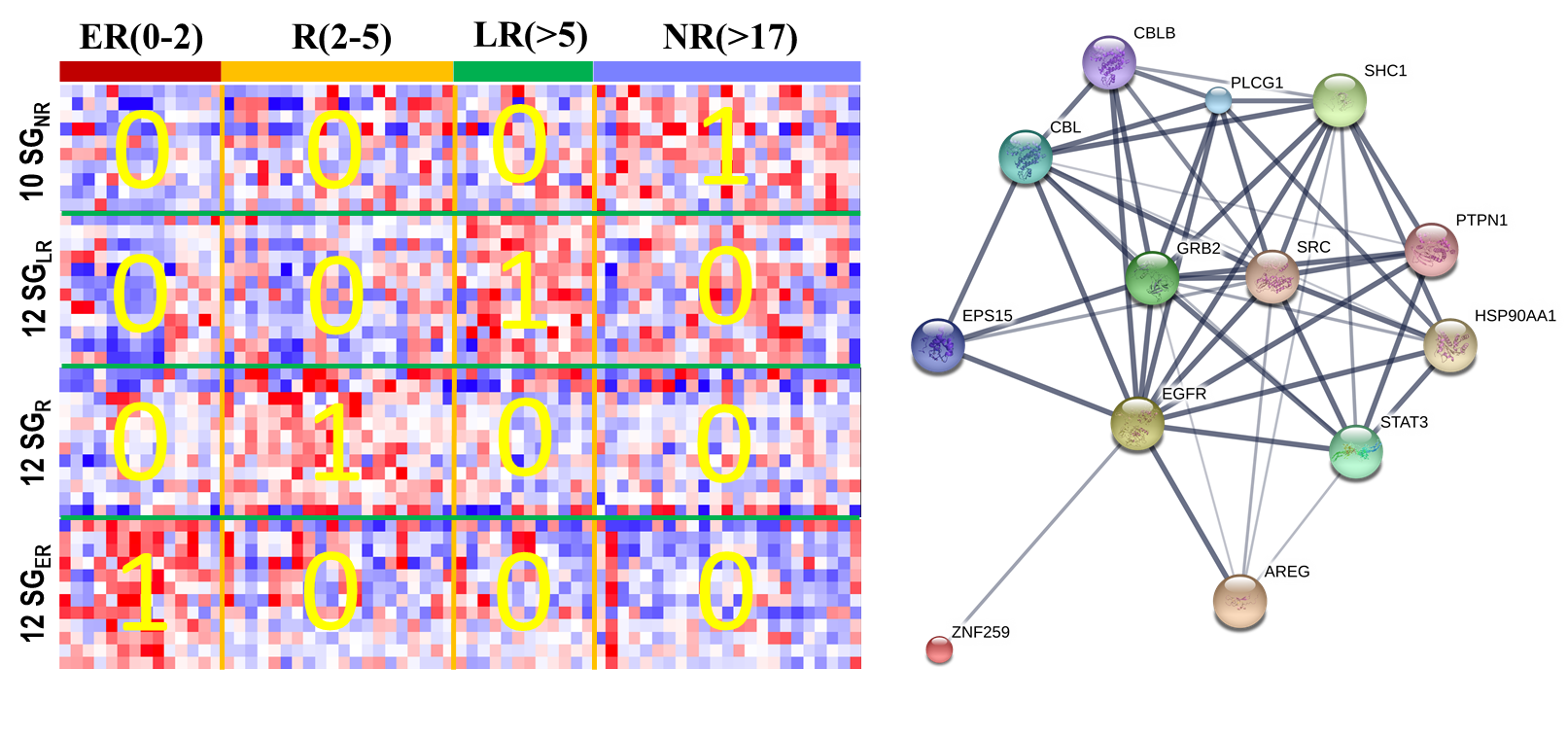
**

**Figure S3**. Signature genes associated with recurrence progression, detected by COT/eCOT and displayed by new heatmap design (Edinburgh breast cancer transcriptomics data), and a typical EGFR-ZNF259-AREG signaling network.

**Table S3**. The protein IDs of SGs associated with NL, FS, and FP, respectively, and their corresponding cosine scores readily provided by the COT tool, on the proteomics dataset acquired from human artery samples enriched by the tissue types associated with atherosclerosis.


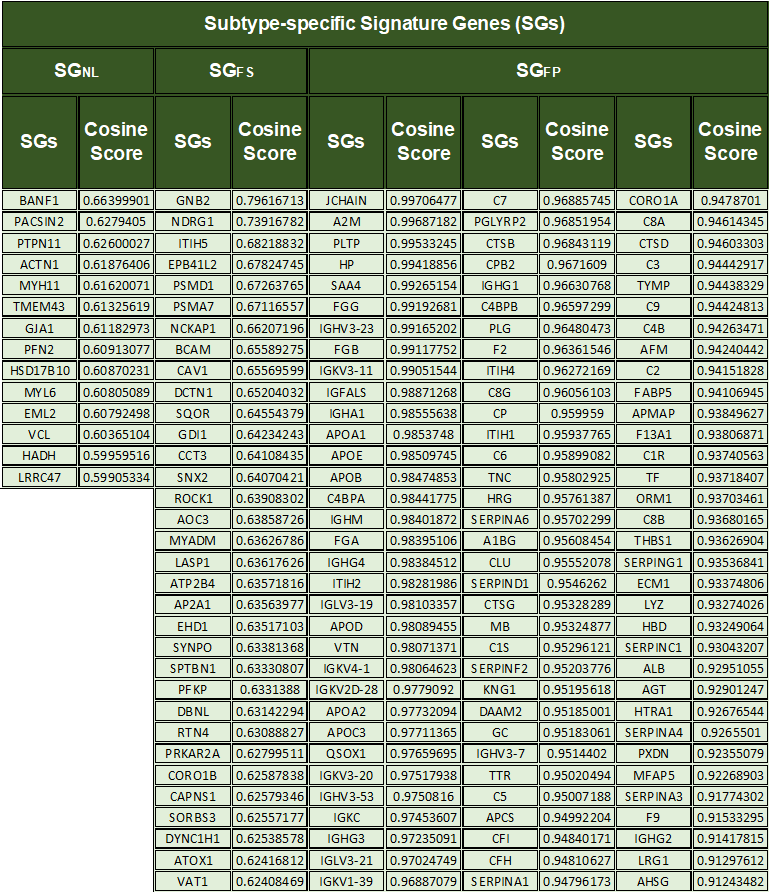


**Table S4**. The protein IDs of DSGs associated with NL, FS, and FP, respectively, and their corresponding cosine scores readily provided by the eCOT tool, on the proteomics dataset acquired from human artery samples enriched by the tissue types associated with atherosclerosis.


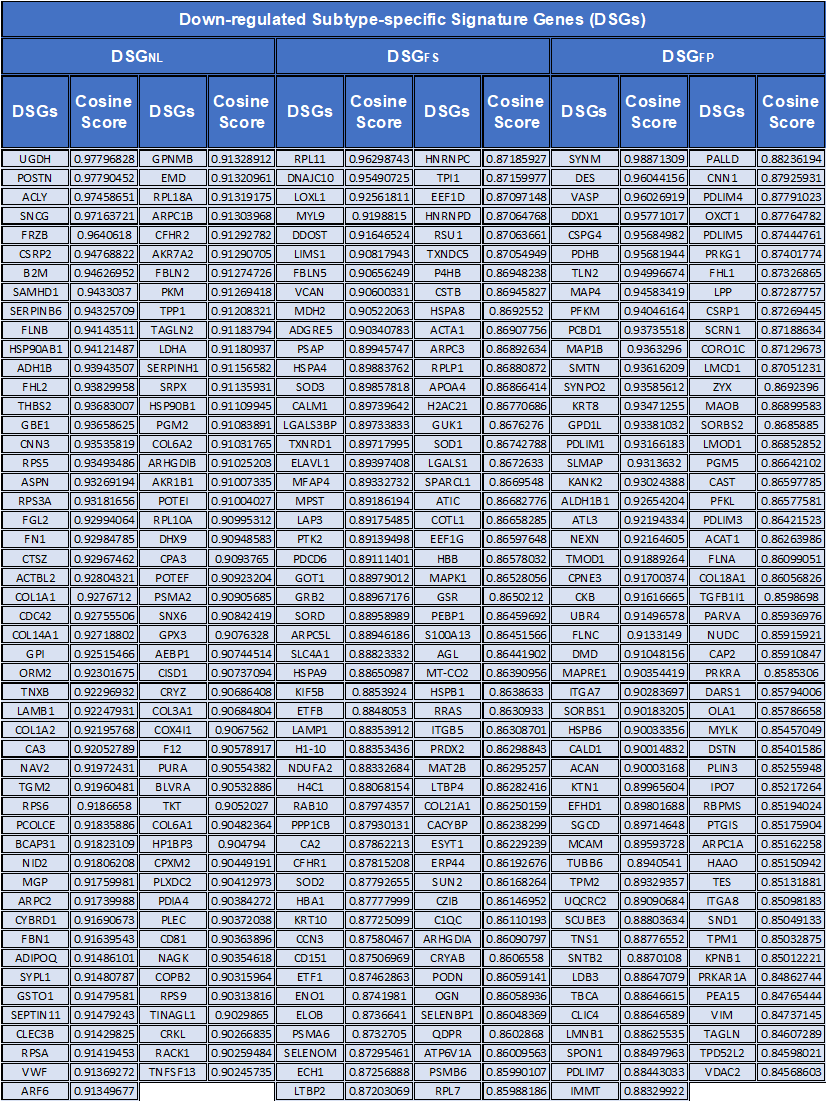


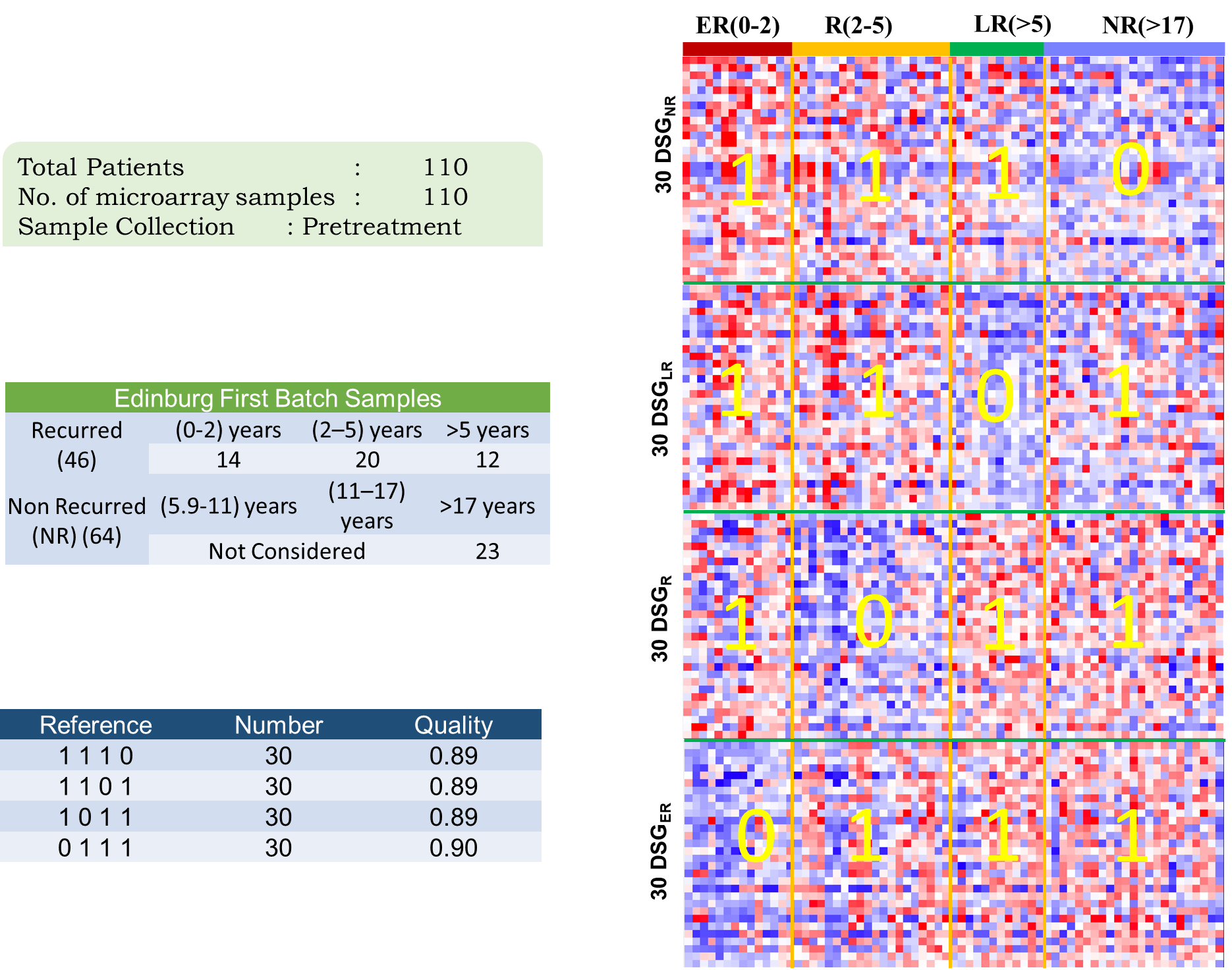


**Figure S4**. The DSGs detected by eCOT on the Edinburgh breast cancer gene expression data that were acquired prior to standard treatment. The DSGs are displayed via new heatmap design and associated with ER (early recurrence, 0~2 years), R (recurrence, 2~5 years), LR (late recurrence, >5 years), and NR (never recurrence, >17 years), respectively.

**Table S5**. The SGs associated with ER, R, LR, and NR, respectively, and their corresponding cosine scores readily provided by the COT tool, on the Edinburgh breast cancer gene expression data that were acquired prior to standard treatment.


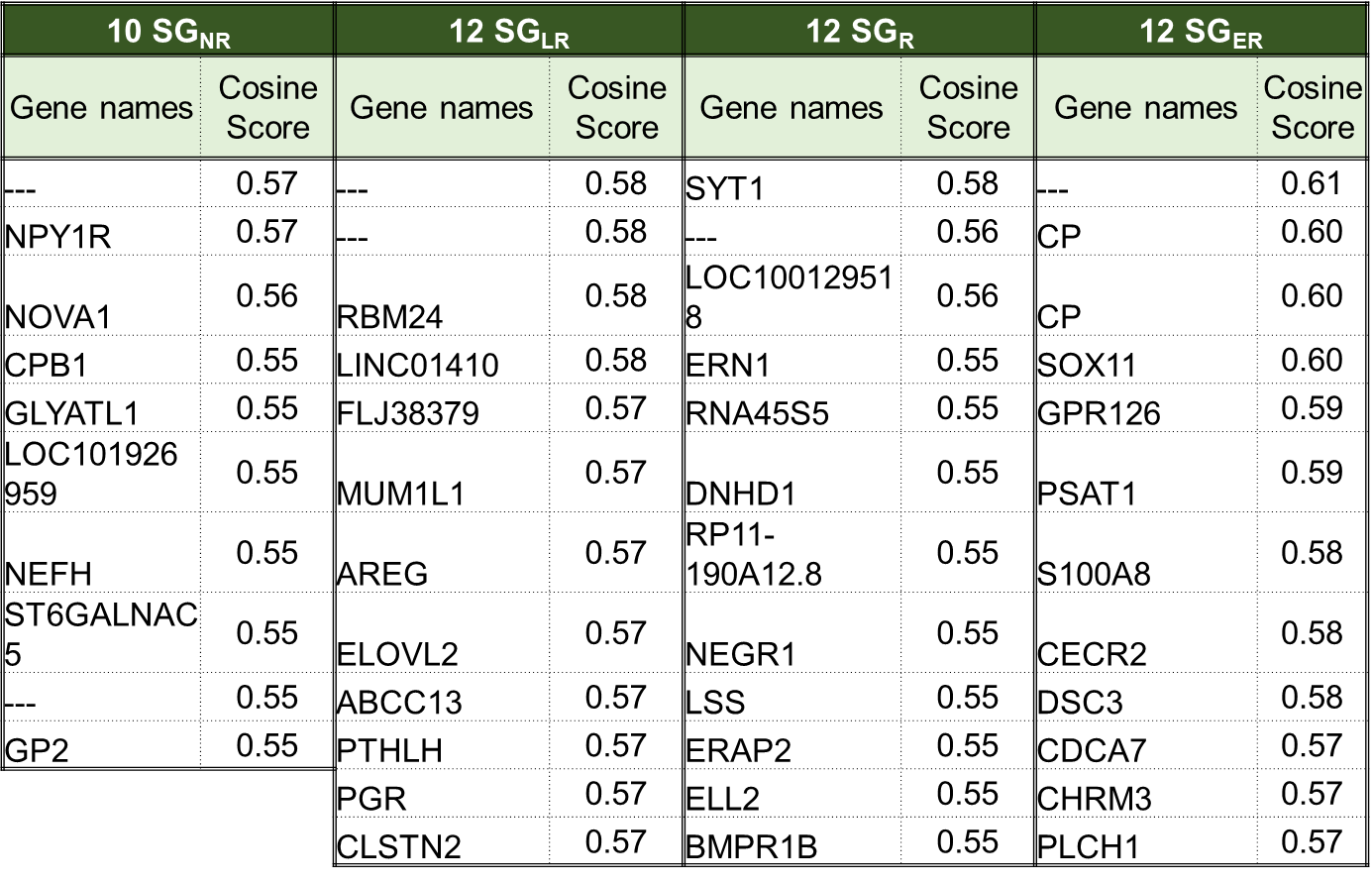


**Table S6**. The DSGs associated with ER, R, LR, and NR, respectively, and their corresponding cosine scores readily provided by the eCOT tool, on the Edinburgh breast cancer gene expression data that were acquired prior to standard treatment.


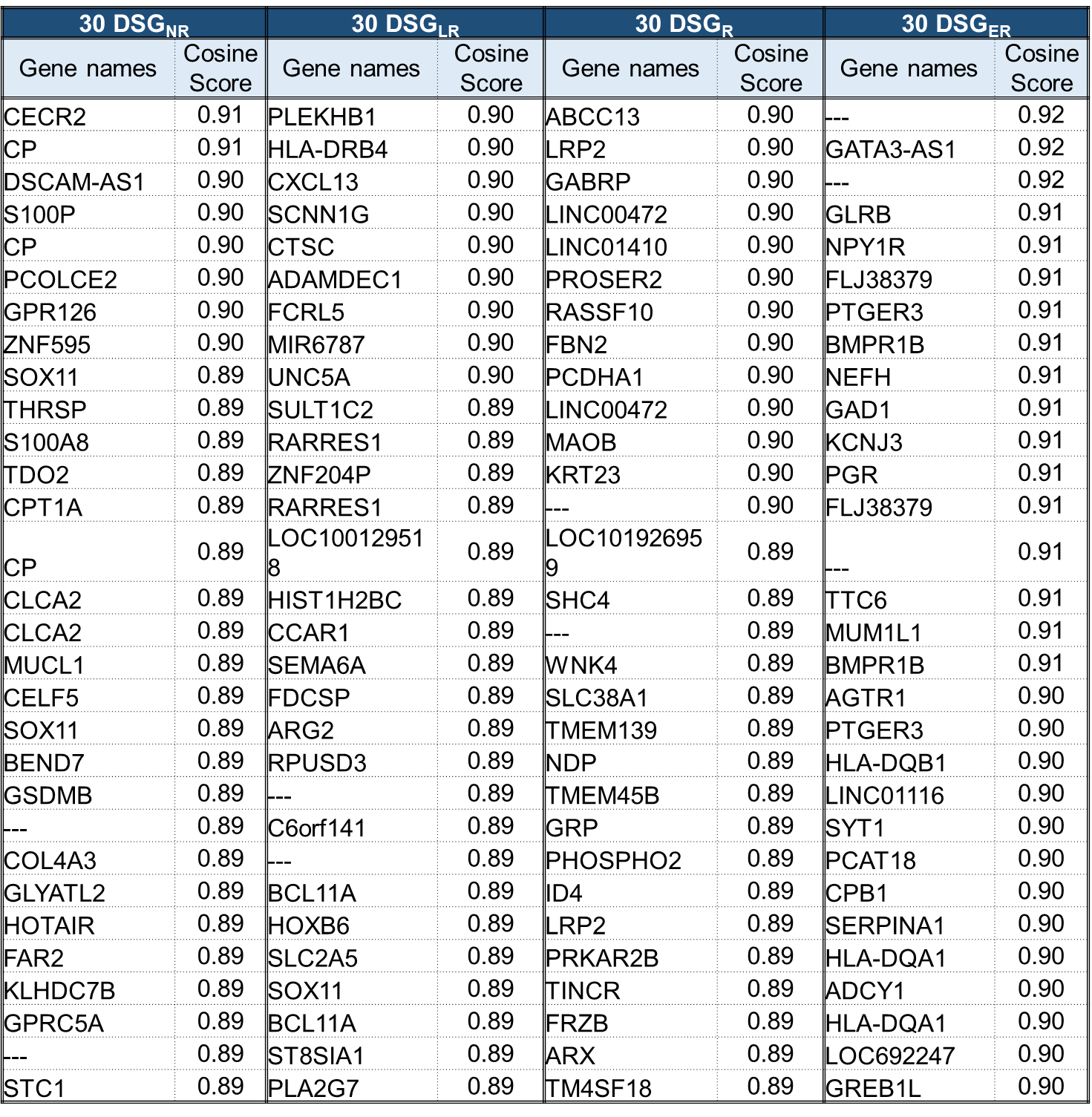


## Discussion

In future work, support vector machine or artificial neural network (ANN) based methods may be considered as emerging imputation competitors (Wang, et al., 2020), and a combination approach utilizing an ensemble of strategies could be explored (Ma, et al., 2020). We have recently begun to explore a deep matrix completion method (Fan, et al., 2021).

## R Scripts

The R scripts are available at <https://github.com/niccolodpdu/ABDS/tree/main>

More suggestions on parameter setting can be found in the package vignette.

## References

Chikina, M., Zaslavsky, E. and Sealfon, S.C. CellCODE: a robust latent variable approach to differential expression analysis for heterogeneous cell populations. *Bioinformatics* 2015;31(10):1584-1591.

Delaney, C.*, et al.* Combinatorial prediction of marker panels from single-cell transcriptomic data. *Mol Syst Biol* 2019;15(10):e9005.

Fan, M.*, et al.* A deep matrix completion method for imputing missing histological data in breast cancer by integrating DCE-MRI radiomics. *Med Phys* 2021.

Kuhn, A.*, et al.* Population-specific expression analysis (PSEA) reveals molecular changes in diseased brain. *Nat Methods* 2011;8(11):945-947.

Lu, Y.*, et al.* COT: an efficient and accurate method for detecting marker genes among many subtypes. *Bioinform Adv* 2022;2(1):vbac037.

Ma, W., al., e. and Wang, P. DreamAI: algorithm for the imputation of proteomics data. *bioRxiv* 2020.

Wang, N.*, et al.* Mathematical modelling of transcriptional heterogeneity identifies novel markers and subpopulations in complex tissues. *Scientific Reports* 2016;6:18909.

Wang, S.*, et al.* NAguideR: performing and prioritizing missing value imputations for consistent bottom-up proteomic analyses. *Nucleic Acids Res* 2020;48(14):e83.
